# Maternal and child reflective functioning in the context of child sexual abuse: pathways to depression and externalising difficulties

**DOI:** 10.3402/ejpt.v7.30611

**Published:** 2016-01-27

**Authors:** Karin Ensink, Michaël Bégin, Lina Normandin, Peter Fonagy

**Affiliations:** 1 École de psychologie, Université Laval, Québec City, QC, Canada; 2 Psychoanalysis Unit, Research Department of Clinical, Educational and Health Psychology, University College London, London, UK

**Keywords:** Child depression, child mentalization, child sexual abuse, externalising difficulties, maternal mentalization, reflective functioning

## Abstract

**Background:**

Sexual abuse is a well-recognised risk factor for child psychopathology. Little is known regarding whether child and maternal mentalization can be considered a potential resource or protective factor in this context, respectively, mediating or moderating the relationship between sexual abuse and psychopathology.

**Objective:**

The aims of this study were (1) to explore the relationships between child and maternal mentalizing, measured as reflective functioning (RF), and child depressive symptoms and externalising difficulties; and (2) to examine whether child mentalizing mediates the relationship between child sexual abuse (CSA) and psychopathology.

**Method:**

A total of 168 children aged 7–12 years and their mothers participated in the study. The sample included 74 dyads where children had experienced sexual abuse. The Child Attachment Interview was rated by using the Child Reflective Functioning Scale to assess children's mentalization, and the Child Depression Inventory was used to assess depressive symptoms. Mothers completed the Parent Development Interview to assess maternal RF and the Child Behavior Checklist to assess their child's externalising difficulties. A model involving direct and indirect paths from CSA, child and maternal RF to child psychopathology was examined using Mplus software.

**Results:**

Child mentalization partially mediated the relationships between CSA and depressive symptoms, as well as the relationship between CSA and externalising difficulties. Maternal mentalization was an independent predictor of child externalising difficulties, with higher maternal RF associated with less externalising difficulties.

**Discussion:**

The findings indicate that by ages 7–12, child mentalization is an important inner resource associated with lower depression and externalising. In addition, this study provides new evidence of the importance of the parent's mentalizing stance for the development of self-regulation and externalising difficulties in both abused and non-abused children. The clinical implications are discussed.

Sexual abuse is a known risk factor for psychopathology, with up to 60% of sexually abused children presenting with moderate to severe symptoms of psychopathology (Kendall-Tackett, Williams, & Finkelhor, 1993; Maniglio, 2009; Putnam, 2003) and over one-third of sexually abused children manifesting clinically significant levels of depressive symptoms (Mathews, Abrahams, & Jewkes, 2013) and externalising difficulties (Tremblay, Hébert, & Piché, 1999). Given that 20% of girls and 5% of boys under the age of 18 experience child sexual abuse (CSA) (Finkelhor, Ormrod, Turner, & Hamby, 2005), it is important to identify and understand putative resilience processes that may have potential implications for therapeutic interventions. Mentalizing is considered to be important in resilience processes. There is evidence that mentalizing mediates the relationship between childhood histories of abuse and symptoms of personality disorder in adulthood (Chiesa & Fonagy, 2014) and moderates the relationship between abuse and parenting outcomes (Fonagy, Steele, Steele, Higgitt, & Target, 1994). This suggests that mentalization may be a potential mediator of risk for depressive symptoms and externalising difficulties in the context of trauma for children too, but no studies have addressed this issue. Furthermore, parental mentalizing is considered to have important implications for the development of self-regulation and affect regulation, but data linking parental mentalizing and child psychopathology remain lacking. The aim of this study was to address the gaps in current knowledge regarding the relationships between children's and maternal mentalization and child psychopathology in the context of CSA.

## Mentalizing

Since the early 1990s, mentalizing has been studied from related, but somewhat different, perspectives—that of theory of mind (ToM) and social cognition, as well as Fonagy and colleagues’ (Fonagy, Gergely, Jurist, & Target, 2002) attachment-based perspective. ToM is a specific dimension of social cognition referring to the capacity to represent mental states such as beliefs, desires, expectations, intentions, and feelings (Happé & Frith, 2014; Premack & Woodruff, 1978); to take the perspectives of others; and to “read the mind” in others’ eyes. Fonagy et al. (2002) focus specifically on mentalizing regarding self and others in the context of close interpersonal relationships, operationalised for research purposes as reflective functioning (RF). They propose a developmental model whereby awareness of mental states emerges in the context of early attachment relationships, in which the child learns to identify and mentally represent his/her own affects through the parent's interest in the child's subjective experience and the parent's pedagogical emotional displays, which focus on the child's mind and feelings. In this model, the parents’ capacity to imagine the subjective experience of their infant/young child is considered to facilitate the development of self-regulation and self-control through efficient use of attention-shifting strategies to regulate distress, as well as representation of and communication about affects (Fonagy, 2004).

### Child sexual abuse and mentalizing

In a recent study, children with a history of CSA were found to have difficulties in mentalizing (measured as RF) and, furthermore, their parents also had lower RF regarding their children and were less able to think about the child's subjective emotional experience (Ensink et al., 2015). This is consistent with previous findings of poorer emotional understanding (Shipman, Zeman, Penza, & Champion, 2000) in children who have been sexually abused, as well as deficits in ToM (Cicchetti, Rogosch, Maughan, Toth, & Bruce, 2003; Pears & Fischer, 2005) and poorer emotional understanding (Edwards, Shipman, & Brown, 2005; Pollak, Cicchetti, Hornung, & Reed, 2000) in maltreated children. There is also evidence that maltreating parents have more difficulty in understanding the affective expressions of their children (Shipman & Zeman, 2001) and engage in fewer emotion-focused discussions with them (Edwards et al., 2005). In family contexts where abuse occurs, some parents may actively undermine the development of mental state thinking in order to avoid engaging with the distress that they cause their children (Allen, Fonagy, & Bateman, 2008; Fonagy & Luyten, 2009). Moreover, from a child's perspective, the experience of abuse and encountering the malevolent intentions of others may contribute to a defensive inhibition of mentalization to reduce anxiety, especially when the abuser is an attachment figure (Allen, 2013; Fonagy, 2004).

### Mentalizing, depressive symptoms, and externalising behaviour difficulties

No studies have addressed the relationship between mentalizing and depression in children, but there is emerging evidence that deficits in mentalizing may be a risk factor for depression in adults. For example, major depressive disorder in adults is associated with lower RF, both generally (Fischer-Kern et al., 2013) and specifically regarding experiences of rejection and loss (Staun, Kessler, Buchheim, Kächele, & Taubner, 2010). There is also evidence of ToM deficits in adults with chronic (Zobel et al., 2010), unipolar (Lee, Harkness, Sabbagh, & Jacobson, 2005; Wang, Wang, Chen, Zhu, & Wang 2008), and remitted (Bora et al., 2005; Inoue, Tonooka, Yamada, & Kanba, 2004; Montag et al., 2010) depression.

There is also a dearth of literature regarding RF and externalising behaviour difficulties. Only one study to date has examined this relationship and found that RF mediated the relationship between a history of abuse and externalising behaviour in adolescents (Taubner & Curth, 2013). In children, externalising behaviour problems have been found to be associated with difficulties in ToM (Hughes & Ensor, 2008) and distorted mentalizing (Sharp, Croudace, & Goodyer, 2008). In addition, low mentalizing by parents, measured using Meins's construct of mothers’ mind-mindedness comments in interaction with infants, has been shown to increase the risk of conduct disorder and oppositional defiant disorder in their children (Centifanti, Meins, & Fernyhough, 2015; Meins, Centifanti, Fernyhough, & Fishburn, 2013). Furthermore, the parents’ mentalizing stance and capacity to consider the child's subjective experience and respond to the child's psychological and attachment needs consequent to trauma can be hypothesised to be crucial for the child to regain a sense of security and trust, to attenuate the biological stress response, and to reduce the negative impact of abuse. When parental mentalization fails and parents are unable to prioritise the child's experience, this can be expected to contribute further to the child's distress and risk of psychopathology.

## This study

The aim of this study was to investigate the relationships between maternal and child mentalizing (measured as RF) and psychopathology and to examine whether child mentalizing mediates the relationships between CSA and child depressive symptoms and externalising difficulties. We hypothesised that (1) CSA would be associated with more depressive symptoms and externalising behaviour difficulties, (2) higher child RF would be associated with lower child depressive symptoms and behavioural difficulties, and (3) higher maternal RF would be associated with lower externalising difficulties. Based on findings from previous research with adults and adolescents, child mentalization may be expected to be a mediator of the relationship between CSA and psychopathology. Using the definitions set out by Rose, Holmbeck, Coakley, and Franks (2004) for developmental research, mediation is present if the link between CSA and child psychopathology is explained by the impact of CSA on children's mentalization.

## Method

### Participants and procedure

Participants were 168 mother–child dyads, comprising 74 dyads (44 girls, 30 boys) where the children had a history of sexual abuse and 94 community control dyads (52 girls, 42 boys). Children were aged 7–12 years and the majority of participants (98%) were Caucasian, reflecting the demographics of the region where the study was conducted.

Children with a history of CSA were referred by health service providers and child protection services in the region. Information regarding CSA was based on medical and social work reports, as well as information from police inquiries. The control group was recruited from health and community services. The aim was to match control group families with families in the abused group on socioeconomic variables and child age (within 6 months). This procedure proved to be effective for child age but there were significant between-group differences in terms of maternal and paternal education and for family income. Because these variables correlated strongly with each other, only maternal education was used as a control variable in further analyses as it was most strongly associated with the outcome variables.

The data reported in this article were collected as part of a larger longitudinal study of risk and protective factors influencing child psychological adjustment and development of mentalization in the context of CSA. Parents and children came to the university clinic, where parents completed the Parent Development Interview and children completed the Child Attachment Interview (CAI), which was rated with the Child Reflective Functioning Scale to assess child RF. Parents also completed the Child Behavior Checklist, used to assess externalising symptoms, and children completed the Child Depression Inventory, used to assess depressive symptoms.

### Measures

#### Child reflective functioning scale

The Child Reflective Functioning Scale (CRFS: Ensink, Target, & Oandasan, 2013) was used to rate videotaped and transcribed data gathered using the CAI (Shmueli-Goetz, Target, Fonagy, & Datta, 2008; Target, Fonagy, Shmueli-Goetz, Schneider, & Datta, 2000). In this 13-question interview, children are asked to give adjectives to describe themselves and their attachment relationships, followed by requests for examples to illustrate why they used these adjectives. For example, children are asked the following questions: “Can you think of three words to describe yourself?” and “Can you think of three words to describe your relationship with your mum?” For each adjective that the child gives, they are asked “Can you give me an example that illustrates why you picked that word, of a time when you were (e.g., generous)?” and “Can you think of a time when your mum got angry with you? Tell me what happened.” This is followed up with probes about why the child thinks the parent reacted in that way and how that made them feel. Children's responses are coded on an 11-point scale (ranging from −1 to 9) in terms of their propensity to understand personal reactions and interpersonal interactions in terms of mental states (see Table 1 for descriptive anchors). A mean score of 5 (simple, but solid mental state understanding) is expected in middle-class samples. Inter-rater reliability of the CRFS items has been shown to be good, with intraclass coefficients (ICCs) ranging from .6 to 1.00, with a median of .93 (Ensink et al., 2013). Temporal stability of children's RF has been shown to be high over a 3-month period and adequate over 12 months (Ensink et al., 2015). In the present study, child RF was rated by the first author and two postgraduate psychology students trained to a criterion of 85% agreement, using a series of training protocols. Inter-rater reliability was calculated on 30% of protocols and was excellent (ICCs ranging from .80 to .90).

**Table 1:** Examples of different levels of child and parental reflective functioning

Rating	Description
−1	Bizarre, disorganised response where mentalizing is actively avoided, or where there is an aggressive refusal to mentalize. Child: “When she gets cross? There is an angel dancing on her shoe.” Parent: “When I am talking on the phone with friends she provokes me by running up and down and the only thing that helps to calm her is to hit her.”
0	Absence of mentalization. Child: “I don't know, it just is.” Parent: “He just does it for no reason, he is just like that.”
1	Descriptions in terms of physical or behavioral non-mental characteristics. Child: “She says—go to your room.” Parent: “He just keeps twirling around, he never stops.”
3	Unelaborated references to mental states when describing relationships. Child: “I like it, it is fun.” Parent: “He gets irritable.”
4	References to mental states, but with gaps that have to be filled in. Child: “When I feel sad, she like … comforts me.” Parent: “When we are preparing for an exam and he messes about and I know it is going to take so much longer, I get so angry.”
5	Clear description showing a solid mental state understanding, even if fairly simple. Child: “When she gets angry, she shouts, and I don't like it, but I know she does not really mean what she says and that I am a little bit to blame.” Parent: “I get angry because he loses everything, his gloves, his books, and when we arrived at school and he had forgotten his gloves again and we had to turn back, and I realized I was going to be late for work, I lost it. But I realize that I need to find a way to help him become more responsible and it does not help to shout.”
7–9	Increasingly sophisticated mental state understanding, with 9 denoting exceptional and complete mental state understanding. Child: “When he gets angry, I also get angry at first, but then I feel guilty, because I know he helps me a lot, and when I forget my books at school it takes much longer, and he gets tired and has work to do too.” Parent: “I don't often get angry with him, but sometimes when he becomes very excited and maybe because he wants to show off I think in front of his friends he behaves in a way that he would not usually, becoming defiant, and I feel a little foolish and frustrated. He does not realize that he actually risks losing his friends’ respect and it makes them feel uncomfortable, and I don't know how to explain without hurting his feelings.”

#### Maternal reflective functioning

Maternal RF was measured using the Parent Development Interview-Revised (PDI-R: Slade, Aber, Bresgi, Berger, & Kaplan, 2004) rated with the accompanying coding manual. The PDI-R is a 45-item semi-structured interview developed to assess parental mentalization regarding themselves, the child, the parent–child relationship, and their relationship with their own parents. The mother is asked for example: “Could you describe (name of the child)?”; “Can you give me an example of a time when he/she was distressed?”; “Describe a time when you became really angry with (name of child)”; and “What effect did this have on him/her?” Reliability estimates using the coding manual have been shown to be good, with ICCs ranging from .78 to .95 (Slade, Grienenberger, Bernbach, Levy, & Locker, 2005). The interview takes approximately 1 hour to complete and is videotaped and transcribed for coding purposes. Demand questions are coded on an 11-point scale using the manual, which provides illustrations of different types and levels of RF responses ranging from −1 (avoidance or active refusal to mentalize) to 9 (exceptionally rich, complete, and sophisticated understanding of mental states in interaction) and where a 5 (clear and solid mental states understanding) is the mean observed in middle-class samples (see Table 1 for descriptive anchors). An overall RF score is assigned following the guidelines in the manual. All protocols were coded by two of the authors of the study, who had been trained to code parental RF. Protocols were allocated so that the same coder never coded both parent and child measures for any dyad. Inter-rater reliability was calculated on 20% of protocols and was satisfactory (ICCs ranged from .67 to .98 and reached .73 for the global PDI-R score).

#### Child Depression Inventory

Based on the Beck Depression Inventory for adults (Beck, Ward, Mendelson, Mock, & Erbaugh, 1961), the Child Depression Inventory is a widely used 27-item child report questionnaire developed by Kovacs (1985) to assess the severity of depressive symptoms in children and adolescents aged 7–17 years. It takes 15–20 min to complete and covers five dimensions including negative mood, interpersonal problems, ineffectiveness, anhedonia, and negative self-esteem. It has been shown to have good psychometric properties, including high reliability and well-established validity. Each item is rated on a three-point scale from 0 to 2, and total scores from 0 to 52 are then converted into standardised T-scores; standardised T-scores of 65 and higher are considered indicative of clinically significant depressive symptoms.

#### Child externalising behaviour difficulties

The Child Behavior Checklist (CBCL-Parent report) is a 118-item questionnaire widely used to assess a broad range of internalising and externalising difficulties. In the present study, the Parent Report Externalizing Scale (which includes aggressive behaviour and rule-breaking behaviour subscales) of the version for children aged 6–18 (Achenbach & Rescorla, 2001) was used. Each item is rated on a three-point Likert scale ranging from *not true* to *very or often true*. The CBCL has been shown to have good psychometric properties (Achenbach & Rescorla, 2001).

### Data analyses

As part of the exploratory analyses a multivariate analysis of variance (MANOVA) was used to compare maternal and child RF, child depression, and externalising difficulties between the CSA and control groups. Exploratory *t*-tests were used to examine possible gender effects as well as possible differences related to whether abuse was intra- or extrafamilial. Correlation analysis was used to examine relationships between maternal and child RF, child depression, and externalising symptoms, and to identify potential control variables.

Next, pathways from sexual abuse to child psychopathology through child RF were examined using a path analysis model in Mplus 7.12 (Muthén & Muthén, 1998–2012). The model tested indirect effects, which involve the same calculations as mediation analyses. Effects tested started from the predictor (abuse) to the outcomes (indices of psychopathology) through child RF as a potential mediator. Furthermore, the potential interaction effects of both child and maternal RF and sexual abuse in explaining child psychopathology were examined. All indirect effects were bootstrapped 1000 times in order to construct bias-corrected 95% confidence intervals (CIs). The bootstrap procedure drew 1000 random samples with replacement of the original sample to construct CIs. Since indirect effects are calculated from the product of unstandardised coefficients, we first standardised all continuous variables in order to see the magnitude of the effects. Path coefficients (*b*) are thus partially standardised indirect effects. Indirect effects are considered significant at the .05 level when the 95% CI does not include the null value 0. Different fit indices were used to test the adequacy of the model: the Comparative Fit Index (CFI), the Tucker–Lewis Index (TLI), the root mean square error of approximation (RMSEA) and the standardised root mean square residuals (SRMR), and the ratio of chi-square to degrees of freedom. Guidelines suggest that values above .95 for the CFI and TLI (Hoyle, 1995) and values below .05 for the RMSEA and SRMR, as well as a non-statistically significant chi-square or ratio of chi-square to degrees of freedom (*χ*^2^/*df*) less than 3, indicate an excellent fit (Browne & Cudeck, 1992; Ullman, 2001).

## Results

### Preliminary analysis

As part of the exploratory analyses, a MANOVA was used to determine whether there were significant differences in maternal RF, child RF, and child psychopathology between children with a history of CSA and children from the comparison group. The results of the MANOVA and the mean and standard deviation for all indicators of child psychopathology and for mother and child RF are reported in Table 2 for children in both groups. Children in the CSA group had a significantly lower level of RF and significantly more symptoms of depression and externalising difficulties, with moderate to strong effect sizes (Cohen's *d*=.75, .71 and .89, respectively). The RF of mothers of abused children was also significantly lower; the effect size was moderate (Cohen's *d*=.56). Mothers of children who had been abused also had fewer years of education; for this reason, maternal education was controlled for in subsequent analysis. There were no gender effects with regard to psychopathology or RF and no differences in relation to whether CSA was intra- or extrafamilial.

**Table 2:** Group comparisons for age, maternal education, maternal and child RF, and child psychopathology in 168 mother–child dyads

Variables	Groups	Mean	SD	*F*
Age	Control CSA	112.06 113.14	17.76 19.80	.136
Maternal education	Control CSA	15.69 13.53	3.58 2.92	17.602*
Child RF	Control CSA	2.84 1.85	1.26 1.39	23.403*
Maternal RF	Control CSA	3.69 2.92	1.36 1.40	12.876*
CDI	Control CSA	46.41 51.77	3.31 9.23	22.377*
CBCL (Ext.)	Control CSA	53.99 64.52	12.04 11.53	32.853*

*Note:* Age (months); maternal education (years); RF, reflective functioning; CSA, child sexual abuse; CBCL (Ext.), Child Behavior Checklist, externalising behaviours; CDI, Child Depression Inventory.

* *p*<.01.

### Relationships between variables

The results of correlational analyses with an alpha threshold of .01 are presented in Table 3. These show that there were significant relationships between child RF and maternal RF, between child RF and depressive symptoms, between child RF and externalising difficulties, and between child RF and age. Maternal RF was significantly related only to child externalising difficulties. Because child RF was moderately associated with age, this was controlled for in further analyses.

**Table 3:** Correlations between age, maternal education, maternal and child RF, and child psychopathology in 168 mother–child dyads

Variable	Age	Maternal education	Child RF	Maternal RF	CDI	CBCL (Ext.)
Age	–	–	–	–	–	–
Maternal education	−.046	–	–	–	–	–
Child RF	.440*	.403*	–	–	–	–
Maternal RF	−.023	.393*	.248*	–	–	–
CDI	−.105	−.304*	−.436*	−.033	–	–
CBCL (Ext.)	−.048	−.231*	−.429*	−.304*	.230*	–

*Note:* Age (months); maternal education (years); CBCL (Ext.), Child Behavior Checklist, externalising behaviours; CDI, Child Depression Inventory. Maternal RF assessed with the Parent Development Interview. Child RF assessed with the Child Reflective Functioning Scale.

* *p*<.01

### Path analysis

**Fig. 1:**
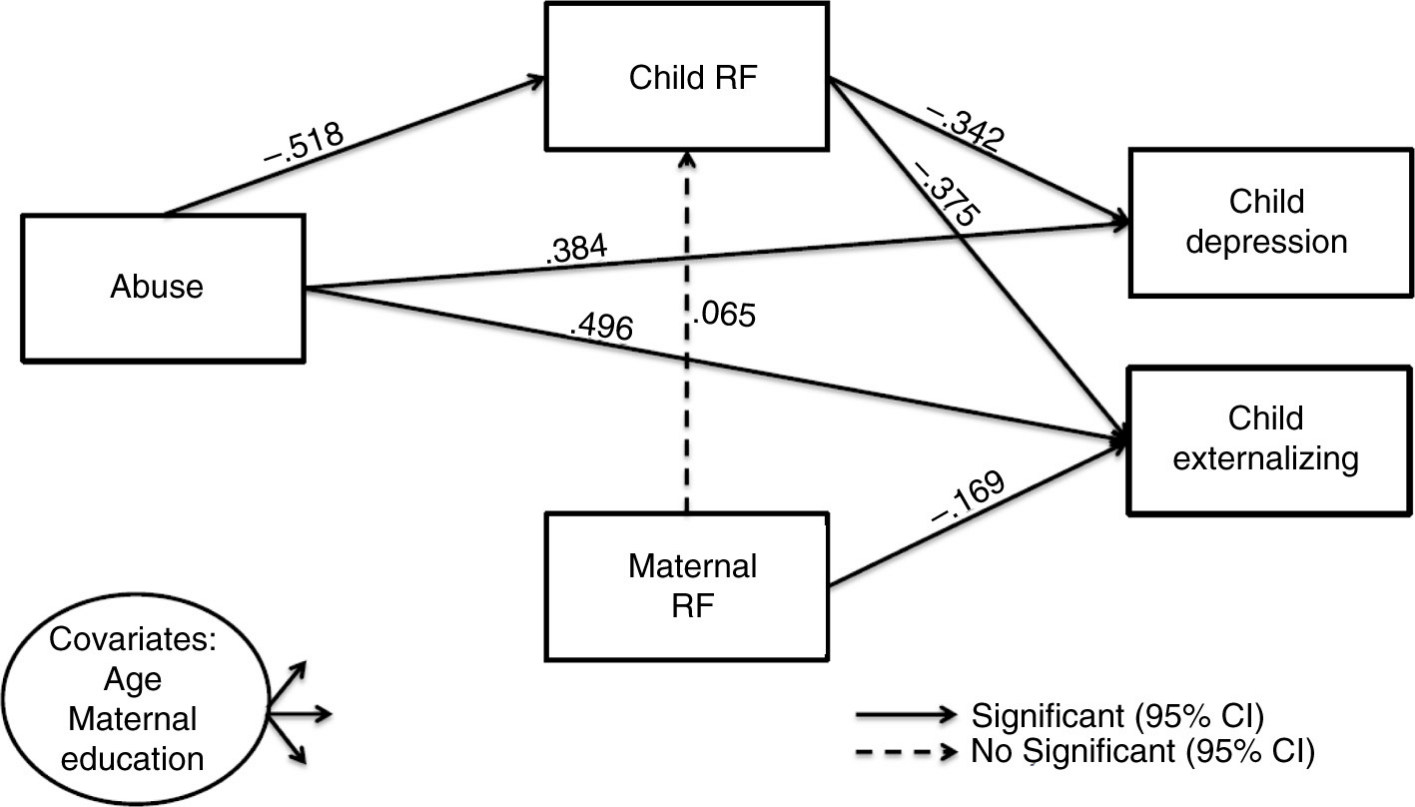
Path analysis describing the associations between child sexual abuse, maternal and child reflective functioning (RF), and child psychopathology (depression and externalising difficulties) in 168 mother–child dyads. *Note*: Child RF assessed with the Child Reflective Functioning Scale; Maternal RF assessed with the Parent Development Interview; Child depression assessed with the Child Depression Inventory; Child externalising assessed with the Child Behavior Checklist. Covariates were allowed to predict all dependent variables in the model (parameter estimates are presented in table 4). Parameters are standardised. CI, confidence interval.

A path analysis was conducted to test the hypothesis that child RF mediated the relationship between CSA and child psychopathology (i.e., depressive symptoms and externalising difficulties). All fit indices showed good to excellent model fit (CFI=.980; TLI=.900; RMSEA=.088; SRMR=.024, and *χ*^2^(3)=6.865, *p*=.08, *χ*^2^/*df*=2.288). A visual depiction of the model is presented in Fig. 1. Parameter estimates are reported in Table 4. Results showed that CSA had a direct effect on child RF (*β*=−.518; *p*<.01). Furthermore, CSA (*β*=.384; *p*<.05) and child RF (*β*=−.342; *p*<.01) had a direct effect on child-reported depression. There was also a significant indirect effect of CSA on depression through child RF (*b*=.177, 95% CI [.081, .285]), which accounted for 31.6% of the total effect. The total effect of CSA on child-reported depression was reduced from *β*=.561 to a direct effect of *β*=.384. This is consistent with partial mediation by child RF of the effect of CSA on child depression. The model explained 24.3% of the variance of child-reported depression.

**Table 4:** Parameter estimates of the associations between abuse, age, mother education, maternal and child RF, and child psychopathology in 168 mother–child dyads

	Estimates	SE	*p*	*R*^2^
Child RF regressed on				.435
Abuse	−.518	.126	.001**	
Maternal RF	.065	.070	.350	
Age	.464	.073	.001**	
Maternal education	.346	.066	.001**	
CDI regressed on				.243
Abuse	.384	.150	.010*	
Child RF	−.342	.089	.000**	
Age	.035	.086	.686	
Maternal education	−.105	.077	.174	
CBCL (Ext.) regressed on				.287
Abuse	.496	.150	.001**	
Maternal RF	−.169	.082	.039*	
Child RF	−.375	.099	.001*	
Age	.110	.084	.191	
Maternal education	.067	.090	.453	

*Note:* Age (months); maternal education (years); CBCL (Ext.), Child Behavior Checklist, externalising behaviours; CDI, Child Depression Inventory. Maternal RF assessed with the Parent Development Interview. Child RF assessed with the Child Reflective Functioning Scale.

* *p*<.05

** *p*<.01.

With regard to externalising behaviour difficulties, the results showed that CSA, lower child RF, and lower maternal RF led to higher levels of externalising. CSA (*β*=.496; *p*<.01), child RF (*β*=−.375; *p*<.01), and maternal RF (*β*=−.169; *p*<.05) had a direct effect on externalising behaviours. Furthermore, CSA had a significant indirect effect on externalising difficulties through child RF (*b*=.194, 95% CI [.079, .363]); after introducing child RF, the direct effect of CSA on externalising difficulties was reduced from a total effect of *β*=.690 to *β*=.496. The indirect effect explained 28.1% of the total effect, indicating that the relationship between CSA and externalising difficulties was partially mediated by child RF. The model explained 28.7% of the variance of externalised behaviours.

## Discussion

The aim of this study was to examine the relationships between child and maternal mentalization and child depressive symptoms and externalising difficulties, as well as to examine whether child mentalization mediates the relationship between CSA and psychopathology. The findings show that by age 7–12, children's mentalization about themselves and their attachment figures was inversely correlated with depressive symptoms and externalising difficulties and that maternal mentalization was inversely associated with child externalising difficulties. Children's mentalization partially mediated the relationships between CSA and psychopathology (depressive symptoms and externalising difficulties), indicating that the link between CSA and psychopathology was partly explained by the negative impact of CSA on children's mentalization. In addition to the expected direct effect of CSA on psychopathology, maternal RF was also an independent predictor of child externalising difficulties.

The finding that children's mentalization partially mediated the relationship between CSA and depressive symptoms is in line with the previous research showing an association between mentalization and depression in adults (Fischer-Kern et al., 2013). Child RF also partially mediated the relationship between CSA and externalising difficulties, in line with Taubner and Curth's (2013) finding that RF mediated the relationship between abuse and externalising behaviour difficulties in adolescents. We found partial rather than full mediation in this study, possibly because we investigated the impact of mentalization more proximal to the experience of CSA, when trauma-related hyperactivation and physiological dysregulation may still be present, whereas in the study of Taubner and Curth (2013) more time had passed since the experience of abuse.

Higher maternal RF was shown to be associated with a lower risk of children aged 7–12 exhibiting externalising behaviour difficulties, extending previous findings of an association between low parental mentalizing and children's later oppositional defiant and conduct problems (Centifanti et al., 2015; Meins et al., 2013). Whereas in previous studies maternal mentalizing was measured using Meins's construct of maternal mind-mindedness manifest in mothers’ verbal interactions with their infants, the findings of the present study show that the association between low maternal mentalizing and externalising difficulties is also evident when maternal mentalization about their primary school-aged children is measured as RF. Parental sensitivity is considered key to the child's acquisition of self-regulation (Sroufe, 2013), and the parent's mentalizing stance has been shown to underlie more sensitive and less negative parental interactions, which are associated with better child outcomes (Ensink, Normandin, Plamondon, Berthelot, & Fonagy, in press). The parent's capacity to look beyond the child's difficult behaviour to his/her subjective experience may help the parent maintain self-regulation, so that the parent does not respond in ways that escalate the child's distress and oppositional behaviour, and may also facilitate communication about the child's frustration and distress that can help the child to understand his/her own reactions and gain self-control. The absence of a similar association between maternal RF and child depressive symptoms suggests that by middle childhood, it is the child's own capacity to mentalize, rather than that of the parent, that has come to have more direct implications for children's internalising symptoms.

In light of the findings of the present study, interventions focused on helping children develop the capacity to communicate about themselves and their relationships in mental state terms may facilitate self-regulation and affect regulation. In addition, improving parental mentalization may be particularly important for helping parents understand their child's subjective experience and see beyond the child's externalising difficulties.

The study has a number of strengths, including the use of objectively rated measures of mentalization for both mothers and children and the inclusion of data regarding both depressive symptoms and externalising difficulties in the context of CSA. However, a number of limitations also need to be considered. While the sample size was sufficient to detect significance and can be considered substantial given the challenges of recruiting children who have been exposed to abuse, the findings need to be replicated with a larger sample. Because of the cross-sectional nature of the study, the direction of the effects tested remains hypothetical. The finding that child externalising difficulties was associated with maternal RF requires replication, given that in this study child externalising difficulties were rated by mothers and it is possible that mothers with low RF perceive more child externalising difficulties. While this study provides new data suggesting that child RF and parental RF may reduce the risk for depressive symptomatology and externalising behaviour difficulties in the context of CSA, further research to examine the relative contributions of other sociocognitive and social information processing difficulties is needed.

## Conclusion

This study provides new evidence that the developing capacity of 7–12-year-old children to think about themselves and others in terms of underlying affects and motivations rather than simply in behavioural terms is associated with better self-regulation and affect regulation for both children with a history of CSA and non-abused children. Children's mentalization partially mediated the relationship between CSA and depressive symptoms and externalising behaviour difficulties. This suggests that mentalization may be an important focus for intervention, both for children in general and especially in the context of CSA. Furthermore, the findings indicate that the parent's mentalizing stance may be particularly important for reducing child externalising difficulties.

## Conflict of interest and funding

There is no conflict of interest in the present study for any of the authors.
